# Depressive symptoms after surgical and medical management of OSA: a systematic review and meta-analysis

**DOI:** 10.1007/s11325-024-03235-6

**Published:** 2024-12-27

**Authors:** Alejandro R. Marrero-Gonzalez, Craig D. Salvador, Shaun A. Nguyen, Ted A. Meyer, Dee W. Ford, Andrea M. Rinn, Chitra Lal, Melissa Swanson, Mohamed Abdelwahab

**Affiliations:** 1 Sleep Surgery Division, Department of Otolaryngology-Head and Neck Surgery, Medical University of South Carolina, 135 Rutledge Avenue, MSC550, Charleston, SC 29425 USA; 2 School of Medicine, University of Puerto Rico, San Juan, Puerto Rico; 3 Department of Pulmonary, Critical Care, Allergy, and Sleep, Medical University of South Carolina, Charleston, SC USA; 4 Expertise Dental, Private Practice, Charleston, SC USA; 5 Department of Oral and Maxillofacial Surgery, Medical University of South Carolina, Charleston, SC USA

**Keywords:** OSA, Sleep surgery, CPAP, Depression

## Abstract

**Purpose:**

To evaluate the effect of CPAP and surgical alternatives for OSA on depression and compare the results of surgery to CPAP.

**​Methods:**

COCHRANE Library, CINAHL, PubMed, and Scopus databases were searched for English-language articles. Meta-analysis of continuous measures (mean), proportions (%), and mean difference (Δ) with 95% confidence interval was conducted for objective and subjective outcomes before and after treatment with CPAP or surgical interventions.

**Results:**

We identified 2018 abstracts, 14 studies (*N* = 3,488) were included in the meta-analysis. Both treatments witnessed significant improvement in Apnea-hypopnea Index (AHI), with similar improvement with CPAP (Δ-48.8 [-51.2, -46.4]) and surgical interventions (Δ -20.22 [-31.3, -9.17]). An improvement in Epworth Sleepiness Scale (ESS) was noted between groups with (Δ -3.9 [-6.2, -1.6]) for the CPAP group and (Δ -4.3 [-6.0, -2.5]) for surgical interventions. The improvement of BDI II depression scores pre- and post-treatment was comparable between treatments with (Δ -4.1 [-5.8, -2.4]) for the CPAP group and (Δ- 5.6 [-9.2, -2.0]) for surgical interventions.

**Conclusion:**

Our findings suggest a reduction in AHI is seen in both CPAP and surgical interventions for OSA, with no difference in AHI reduction between groups. Both treatments also lead to a similar improvement in depression scores providing strong evidence regards impact of surgery on OSA-associated mood disorders. While percent reduction in depression is higher in the surgical group, the difference did not reach statistical significance when compared to CPAP. When stratified by surgical intervention, most interventions suggest an improvement in depression scores.

**Supplementary Information:**

The online version contains supplementary material available at 10.1007/s11325-024-03235-6.

## Introduction

Obstructive sleep apnea (OSA) is the most common sleep breathing disorder involving repeated blockage or cessation of airflow during sleep [1]. Patients with OSA are at increased risk of health-related conditions and reduced quality of life [2]. Factors that contribute to the reduction in quality of life in OSA include poor sleep, insomnia, memory loss and cognitive impairment [3]. Depression is also associated with poor sleep quality, fatigue, and cognitive decline. Clinical studies have shown that the prevalence of depression is up to 66% among patients with OSA [4], that could be contributed to sleep loss, excessive daytime sleepiness, and cognitive changes mentioned [5]. 

Different treatment modalities exist to manage OSA, from non-invasive medical interventions to surgical procedures. Continuous positive airway pressure (CPAP) is the first-line treatment for OSA and is effective for most phenotypes. Studies have shown that the use of CPAP reduced excessive daytime sleepiness and adverse events related to other medical conditions [6, 7]. However, some patients experience nasal airway problems, mouth leakage, discomfort wearing masks, and aerophagia, making CPAP difficult to tolerate [8]. For such patients who struggle with CPAP, other options, such as surgical interventions, exist and provide comparable results and, sometimes, superior clinically relevant outcomes [9, 10]. Examples of surgical treatment options include tonsillectomy, hypoglossal nerve stimulation (HNS), uvulopalatopharyngoplasty (UPPP), maxillomandibular advancement (MMA), etc [11–13]. Some studies have looked into the effect of individual surgical treatment of OSA in depression, but no consensus about the effect of surgical interventions on depressive symptoms exists, especially in comparison to CPAP [11, 12]. We hypothesize that surgery may have similar effects on polysomnographic data and depression scores as CPAP.

This systematic review aimed to comprehensively examine the effects of CPAP and surgical procedures on depressive symptoms in adults with OSA. We hypothesized that the surgical treatment options are comparable to CPAP in reducing depression in patients with OSA.

## Methods

### Information source and search strategy

This study was conducted according to the Preferred Reporting Items for Systematic Reviews and Meta-Analyses (“PRISMA”) guidelines [14]. Detailed search strategies were developed for Cochrane Library (Wiley), PubMed (National Library of Medicine – National Institutes of Health), SCOPUS (Elsevier), and CINAHL (EBSCO). Databases were searched from inception through August 1, 2023, with the results limited to English language articles. A combination of subject headings (e.g., Medical Subject Headings [MeSH] in PubMed) and the following keywords were used in the search: “Obstructive Sleep Apnea” or “OSA,” “Depression,” “Major Depressive Disorder” and “Depressive symptoms” The complete list of search terms is available in Table S1. Articles were analyzed using the review management software Covidence (Veritas Health Innovation Ltd). Titles and abstracts were screened for relevance, and full texts were reviewed to determine inclusion. References of all included articles were examined for additional studies.

### Selection criteria

The following study designs were considered for inclusion: cohort studies, retrospective and prospective case series, randomized control trials, and case-control studies. Studies that measured the pre- and post-treatment depression patient-reported outcomes measures questionnaire (PROMS) scores were selected as shown in Table S2. The exclusion criteria included review articles, studies with incomplete data, case reports, studies with incorrect study design, and studies that did not measure the outcome of interest. Two reviewers (A.R.M and C.D.S.) independently screened titles and abstracts to identify all relevant articles, after which the full texts of these articles were examined to determine which articles would be included in the final analysis. A third reviewer resolved any conflicts between reviewers.

### Data collection process and data items

The reviewers independently extracted data and compared results for accuracy. The third reviewer resolved any conflicts between reviewers. Data extracted from studies included author, publication year, patient demographics (i.e., age, sex, etc.), type of treatment, Apnea-Hypopnea Index (AHI), BDI-II, Epworth Sleepiness Scale (ESS) score, etc.

The BDI questionnaire is a 21-item self-report questionnaire designed to measure the presence and severity of depressive symptoms, with scores ranging from 0 to 63 [15, 16]. Each item is rated on a 4-point scale, with higher scores indicating more severe symptoms. This tool captures various aspects of depression, including sadness, loss of pleasure, and fatigue, and is widely used for its reliability and validity in both clinical and research settings.

The ESS questionnaire is a self-administered scale that assesses the propensity for daytime sleepiness across eight common situations, such as sitting quietly or watching TV. Participants rate their likelihood of dozing off on a 0 to 3 scale for each scenario, with total scores ranging from 0 to 24. A score greater than 10 typically suggests excessive daytime sleepiness, which is often associated with conditions like obstructive sleep apnea [17]. 

### Critical appraisal

Included articles were critically appraised to assess the level of evidence using the Oxford Center for Evidence-Based Medicine criteria [18]. The risk of bias was assessed according to the Cochrane Handbook for Systematic Reviews of Interventions version 6.1 [19]. Since the included studies were a mix of randomized clinical trials or retrospective reports, Version 2 of the Cochrane risk-of-bias tool for randomized trials (RoB 2) and the Risk of Bias in Non-Randomized Studies–of Interventions (ROBINS-I) tool were used [20, 21]. Bias due to confounding, bias in the selection of participants into the study, bias in the classification of interventions, bias due to deviations from intended interventions, bias due to missing data, bias in the measurement of outcomes, and bias in the selection of the reported results were the risk of bias items assessed. The two reviewers independently performed risk assessments on all studies. The risk of bias for each aspect was graded as low, unclear, or high.

### Data analysis and synthesis of results (statistical analysis)

Fourteen studies were synthesized and included in metanalysis in Table 1. Studies that were chosen included pre- and post-BDI-II scores. This depression scale was chosen because of its wide utility amongst both CPAP and surgical interventions in the literature.

**Table 1:** Descriptive features of included studies in the systematic review

Author (yr)	Country of study	OCEBM level of evidence	Total patients (*n*)	Male (*n*)	Age (M)	Age (SD)	Treatment	Follow-up
Alemohammad 2022	Iran	3	28	11	51.2	10.9	CPAP	3–12 months
Balsevičius 2015	Lithuania	3	19		46	5.75	UPPP	3 months
Bouloukaki 2014 A	Greece	2	1550	1193	55.6	10.2	CPAP	24 months
Bouloukaki 2014 B	Greece	2	1550	1133	55.1	10.7	CPAP	24 months
Castronovo 2009	Italy	4	14	14	43.93	7.78	CPAP	3 months
Eastwood 2011	USA	2	21	14	53.6	9.2	HNS	3 months
Habukawa 2010	Japan	3	17				CPAP	2 months
Ishman 2014	USA	3	44	32	44	10.2	Multiple Surgery	3 months
Kaminska 2018	Canada	3	21	17	66.4	10.1	CPAP	12 months
Lee 2017 A	Taiwan	3	36	32	61.9	11.2	CPAP	6 months
Lee 2017 B	Taiwan	3	43	35	48.1	13.3	CPAP	6 months
Lin 2020		3	53	40	35.66	11.66	MMA	12 months
Li 2016 A	Taiwan	4	10	10	37.3	7.18	UPPP	1 month
Li 2016 B	Taiwan	4	29	23	47.69	13.56	CPAP	1 month
Maniaci 2022	Germany	3	20	16	43.35	7.82	Pharyngoplasty	6 months
Vanek 2023	Czechia	3	81	56	54.9	9.9	CPAP	2 months

*M *mean, *CPAP *Continuous Positive Airway Pressure, *UPPP *Uvulopalatopharyngoplasty, *HNS *Hypoglossal Nerve Stimulation, *MMA *Maxillomandibular advancement surgery

Meta-analysis of single means (age, AHI, ESS, baseline BDI Depression PROMs) was performed by Comprehensive Meta‐Analysis version 4 (Biostat Inc, Englewood, NJ, USA). Meta-analysis of continuous measures (AHI, BDI-II, ESS) between pre-treatment vs. post-treatment groups was performed with RevMan v5.4.2 (The Cochrane Collaboration, 2020). Each measure (mean (M) /mean difference (Δ) and 95% confidence interval (CI) was weighted according to the number of patients affected. Heterogeneity among studies was assessed using χ2 and I^2^ statistics with fixed effects (I^2^ < 50%) and random effects (I^2^ > 50%). A Z-test was conducted to evaluate the statistical significance of the differences in percentage reductions of AHI and BDI-II scores between CPAP and surgery. For the non-inferiority analysis of BDI-II percent reduction between CPAP and surgical interventions, we conducted Z-test using a predefined non-inferiority margin. We tested whether surgical interventions were non-inferior to CPAP, assessing statistical significance at margin established by literature of 17.5% for BDI-II [22].Finally, potential publication bias was evaluated by visual inspection of the funnel plot and Egger’s regression test, which statistically examines the asymmetry of the funnel plot [23, 24]. A *p* value of < 0.05 indicated a significant difference for all statistical tests.

## Results

### Study characteristics

**Fig. 1:**
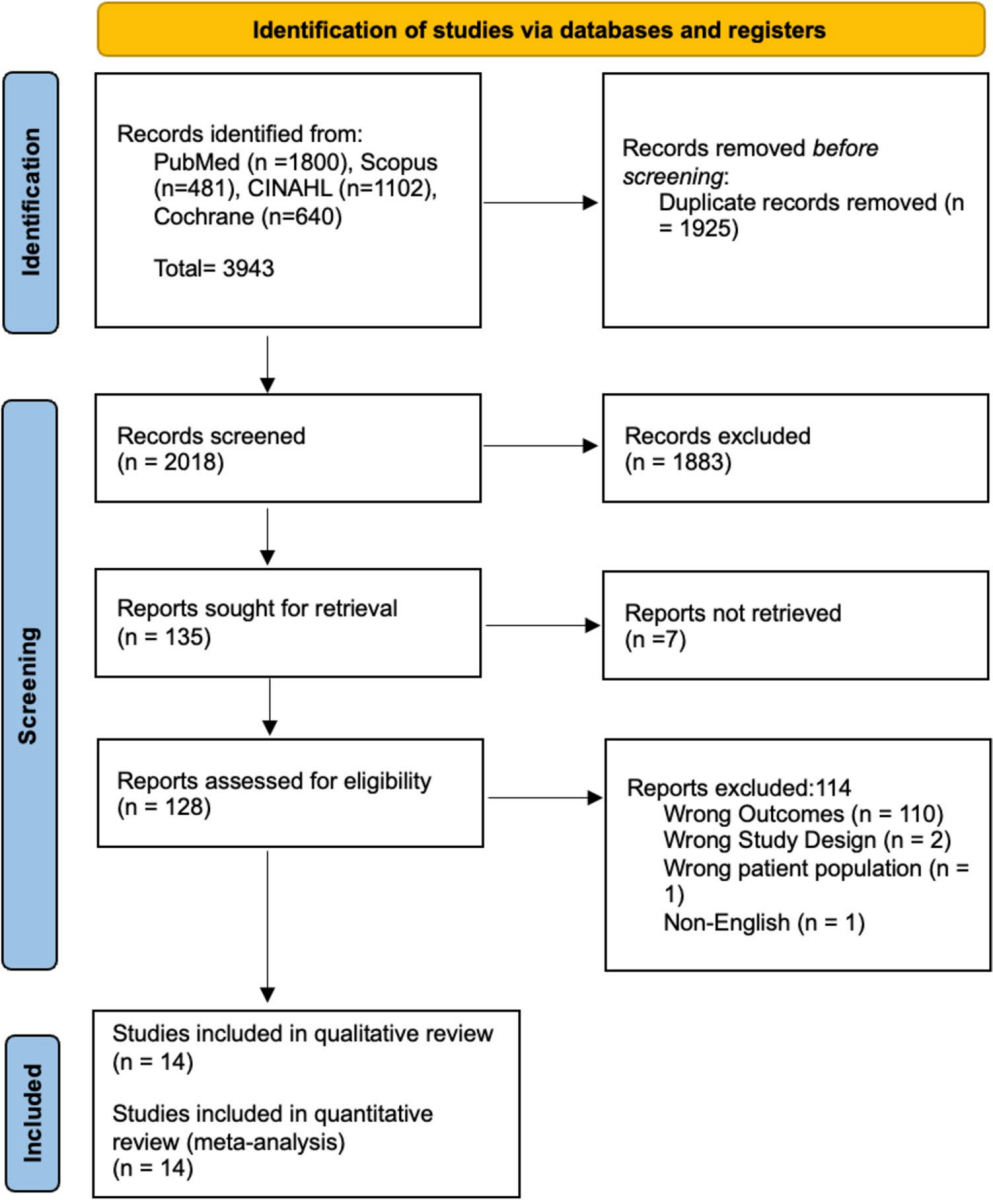
Preferred rEporting Items for Systematic Reviews and Meta-analyses (PRISMA) diagram

After conducting a thorough literature search, we found 2018 unique articles. We then screened the titles and abstracts of these articles and excluded 1883. This resulted in 135 studies assessed in full text. After careful consideration, we included 14 articles in the systematic review and meta-analysis. The search and screening process is in Fig. 1, which shows the PRISMA diagram. The studies we included were level 2 to level 4 based on the Oxford level of evidence and were published from the database’s inception to August 1, 2023. We have summarized the descriptive features of the included studies in Table 1.

Critical appraisal of studies indicated an acceptably low risk of bias for the majority of included studies. Potential sources of bias in non-randomized studies with ROBINS-I (Fig. S1) were most pronounced due to confounding, selection of participants, and missing data. The majority of randomized studies in ROB 2 (Fig. S2) were considered low risk, with greater potential for bias in regard to blinding of participants and blinding of outcome assessment. A funnel plot with Egger’s test (−0.01, 95% CI −0.96 to 0.95, p =. 99) indicated that all studies were within the funnel except for four, suggesting little publication bias.

This study compared CPAP and surgical interventions using three measurable outcomes: AHI, ESS, and BDI-II. Additional stratified analysis for surgical interventions was done for each BDI for depression scores.

### Apnea-hypopnea index

**Fig. 2:**
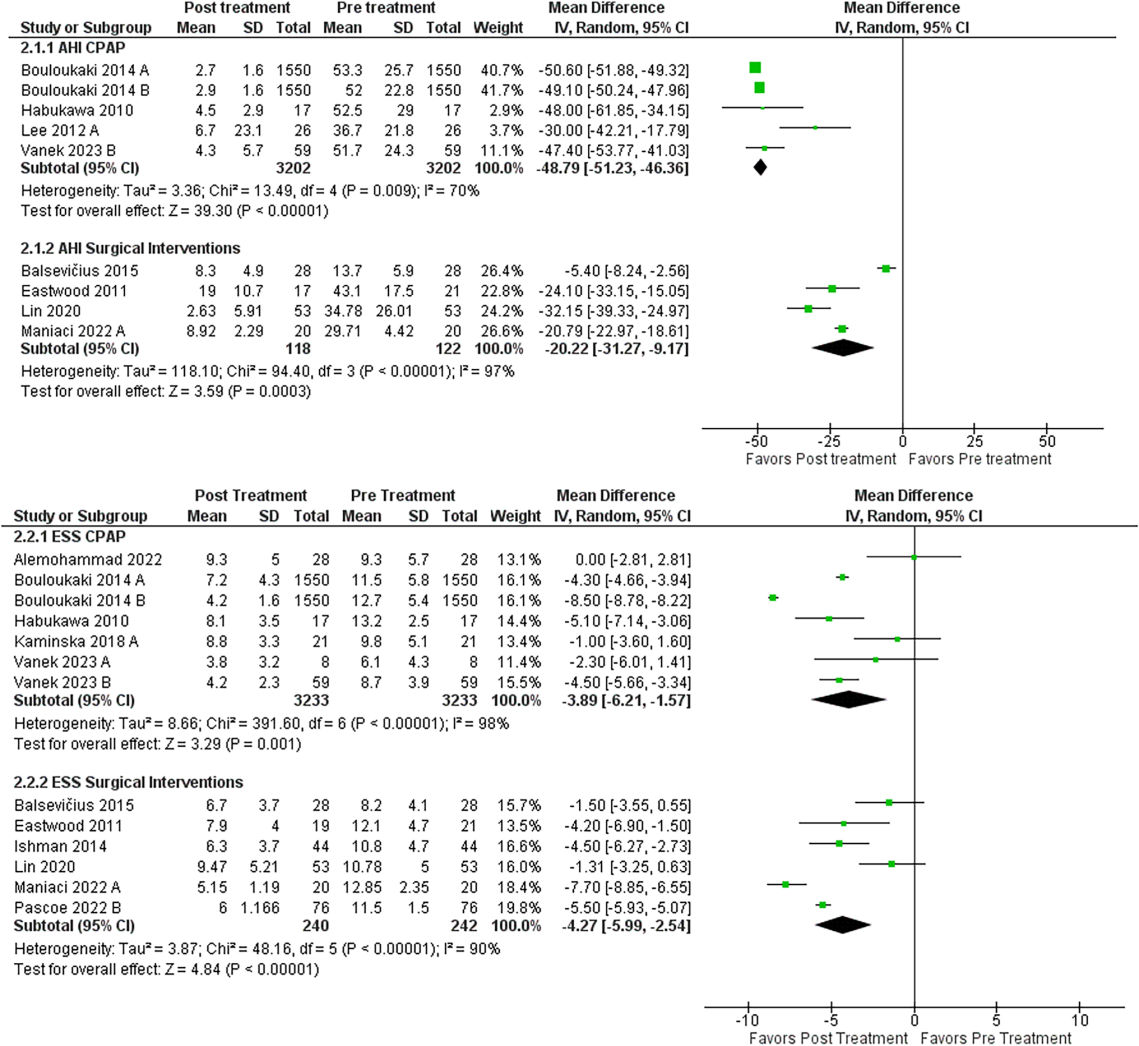
Forest plot of mean difference (MD) on the Apnea-Hypnea Index (AHI) and the Epworth Sleepiness Scale (ESS) from pre and post-treatment. Meta-analysis of AHI. The top panel shows CPAP mean differences in AHI. The bottom panel shows the mean differences in surgical intervention in AHI. CI = confidence interval; IV = inverse variance; CPAP = contnuous positive airway pressure; SD = standard deviation

Thirteen studies containing 17 individual treatment arms reported both pre- and post-treatment AHI for 3488 patients. The mean pre-treatment AHI for studies that used CPAP as treatment was M = 51.3 [48.7, 53.9]. For the studies that used surgery as treatment, a wide array of surgeries were used, such as MMA, UPPP, HNS, and pharyngoplasty (Table 1). The mean pre-treatment AHI of the surgery group AHI was M = 29.9 [18.3, 41.6]. In Fig. 2, AHI was reduced among the CPAP treatment modality; Δ−48.8 [−51.2, −46.4] between pre- and post-treatment. The percent reduction in AHI for the CPAP group was − 95.1% [−95.3%, − 94.8%]. On the other hand, the surgical intervention group had an AHI reduction of Δ−20.2 [−31.3, −9.17]. For this group, the percent reduction for AHI was − 69.9% [−88%, −51.8%]. There was no significant difference in AHI reduction between both groups (*p* > 0.05).

### Epworth sleepiness scale

Eleven studies containing thirteen individual treatment arms reported both pre- and post-treatment ESS for 3475 patients. The mean pre-treatment ESS for studies that used CPAP as treatment was M = 10.7 [9.6, 11.8]. For the surgical treatment modality studies, the mean pre-treatment ESS was M = 11.1 [10.1, 12.1]. In Fig. 2, an improvement between pre- and post-treatment ESS was seen between groups with Δ −3.9 [−6.2, −1.6] for the CPAP group and Δ −4.3[−5.9, −2.5] for surgical interventions. For the CPAP group, the percent reduction between pre- and post-treatment ESS was − 36.5% [−57.9%, −14.9%] compared to the surgery group, which had a percent reduction of −38.7% [−53.2%, −22.5%].

### Beck depression inventory II

**Fig. 3:**
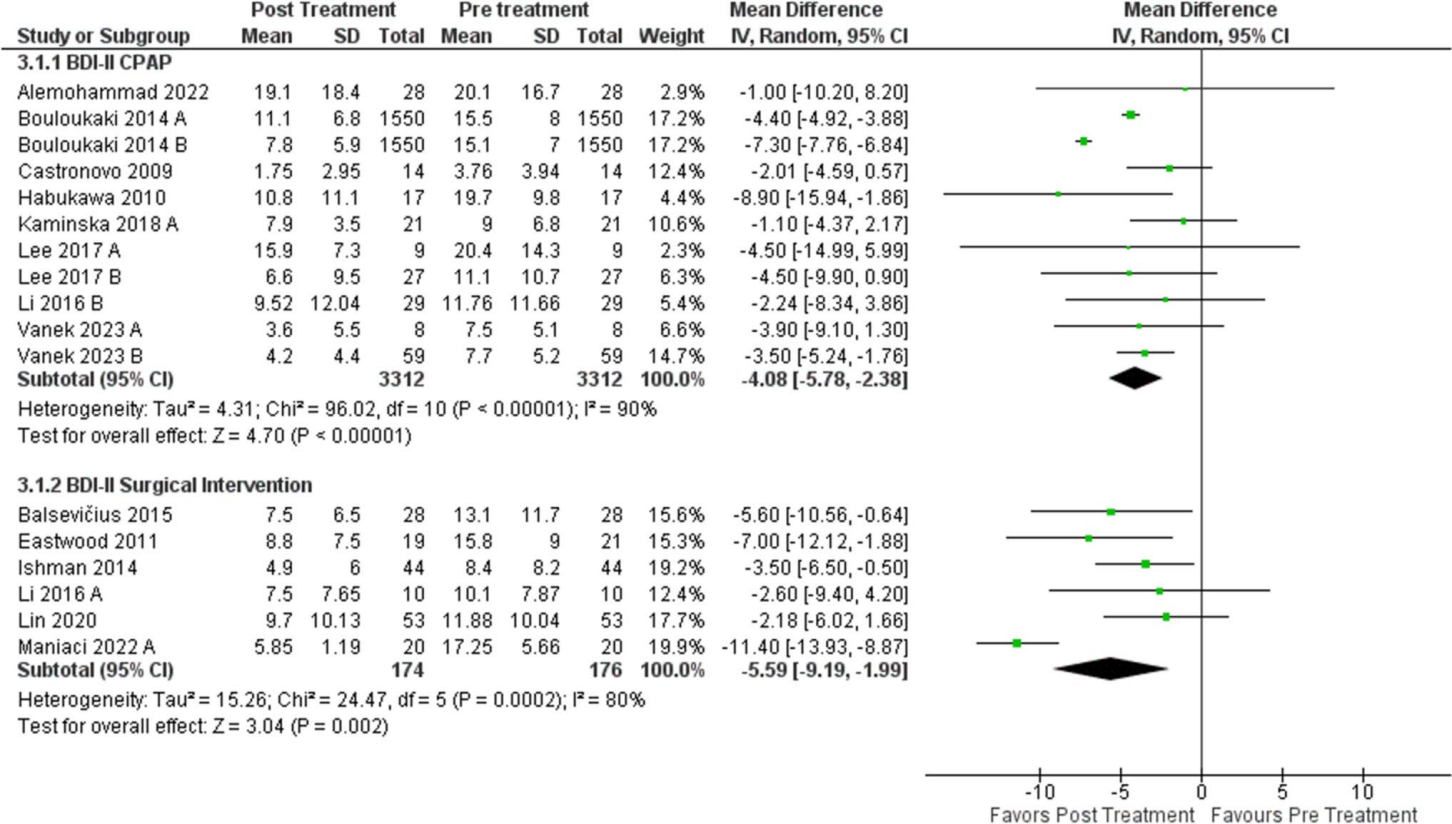
Forest plot of mean difference (MD) on the Beck Depression Index II (BDI-II) from pre and post-treatment. Meta-analysis of BDI. The top panel shows CPAP mean differences in BDI-II. The bottom panel shows surgical intervention mean differences in BDI-II. CI = confidence interval; IV = inverse variance; CPAP = continuous positive airway pressure; SD = standard deviation

Fourteen studies containing seventeen individual treatment arms reported BDI-II depression PROM scores before and after treatment. A total of 3488 patients were included. The mean pretreatment BDI-II was M = 11.9 [9.9, 13.9] for the CPAP group and M = 12.7 [9.6, 15.9] for surgical interventions. An improvement of BDI-II scores pre- and post-treatment was comparable between treatment modalities with (Δ −4.1 [−5.8, −2.4]) for the CPAP group and (Δ- 5.6 [−9.2, −2.0]) for surgical interventions seen in Fig. 3. The percent reduction in BDI-II for patients in the CPAP group was − 34.5% [−48.7%, −20.1%]. For the patients in the surgical treatment group, the percent % reduction for BDI-II was − 44.0% [−72.4%, −15.8%]. Although the percent reduction suggested a greater reduction in depression scores with surgery, when compared to CPAP, this difference did not reach statistical significance (*p* = 0.55). With the non-inferiority margin set to 17.5% [22], surgical intervention is non-inferior to CPAP in reducing BDI-II scores with a Z-score = 1.68, and *p* = 0.047.

### Surgical interventions BDI-II sub-analysis

**Fig. 4:**
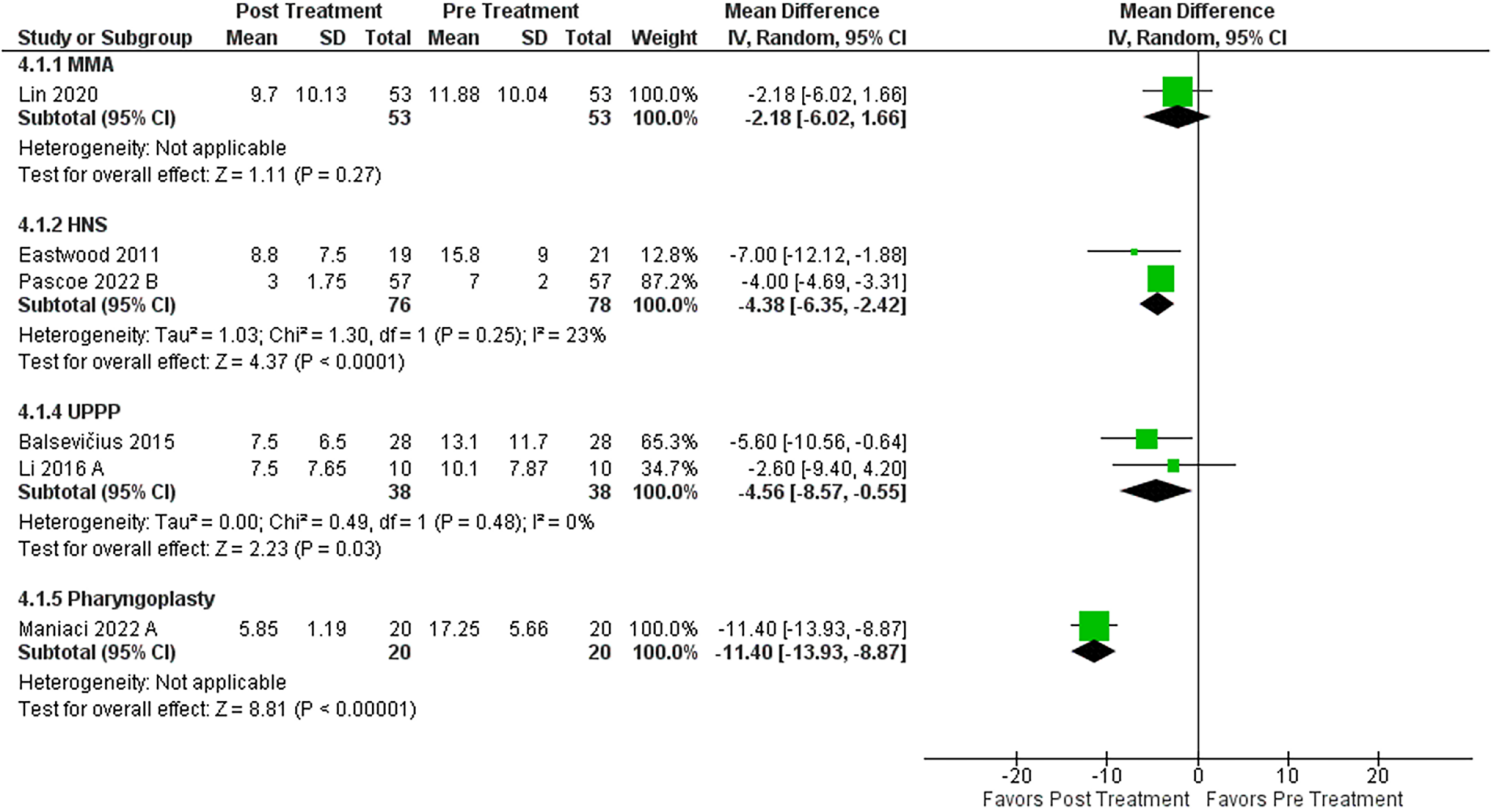
Forest plot of mean difference (MD) on the Beck Depression Index II (BDI-II) from pre and post-treatment stratified by surgical modality. Meta-analysis of BDI; CI = confidence interval; IV = inverse variance; CPAP = continuous positive airway pressure; SD = standard deviation

Seven studies, including 4 different surgical techniques, were analyzed. A total of 217 patients were included in this analysis as shown in Fig. 4. It is also critical to highlight that on categorizing surgical procedures individually, we found significant improvement in depression scores after UPPP, and most recently HNS (*p* < 0.05).

## Discussion

The relation between OSA and mood disorders has been clearly described [4, 25], highlighting an association between depression and OSA [26]. Several proposed mechanisms link OSA and depression, including a disrupted sleep architecture and intermittent episodes of hypoxemia, which can cause an imbalance in neurotransmitters that impair various neuropsychological and affective domains such as mood [27–29]. Previous studies have evaluated the impact of treatment of OSA on mood symptoms, but results indicated a high level of bias in the studies used [30]. Therefore, there is a gap in knowledge about the influence of treatments for OSA on mood symptoms. Previous systematic reviews have tried to address the role of CPAP in depressive symptoms in patients with OSA [31, 32], but their results were limited [26]. We synthesized data from approximately 3488 participants, and we explored the effect of OSA treatments on depression. Our SR-MA concluded that improvement in PROMs depression score is seen in the CPAP and the surgery groups as well as compared its impact to CPAP therapy on depression in adults.

Our focus was on the effect of treatments on depression. Our study also examined how these treatments helped improve polysomnographic metrics in OSA patients. Previous studies have used a reduction of AHI as one of the metrics for treatment efficacy [33, 34]. Our meta-analysis showed a significant reduction in AHI in both treatment groups. When we investigated the percent reduction in our study, a bigger reduction was seen in the CPAP group, but it was not significantly different. CPAP remains the gold standard in managing symptoms in OSA, demonstrating a more pronounced improvement in AHI than with surgical interventions. This reduction in AHI supports previous findings from SR-MA about the efficacy of CPAP over other treatments in OSA patients, yet tolerance is a big pitfall [35]. However, other non-randomized studies that compared the efficacy of surgical intervention versus CPAP have found that surgical interventions might result in a bigger reduction in AHI [36, 37]. In a randomized clinical trial by MacKay et al., reported that patients who underwent combined palatal and tongue surgery had reduced number of apnea and hypopnea events and ESS scores at 6 months when compared to patient with medical management [36]. We have also shown the superiority of surgery in health care utilization; [10] including inpatient, outpatient services and pharmaceuticals. Similarly, while CPAP and surgery groups improved clinically relevant outcomes, surgery was shown to have a superior benefit in the long term, which could be attributed to compliance [9]. It is crucial to acknowledge the variability in surgical interventions encompassed in our study, ranging from UPPP to HNS and MMA. The heterogeneity in surgical techniques likely contributes to the observed differences in AHI outcomes. It is crucial to identify different OSA phenotypes when considering various surgical options. Surgical interventions significantly reduce AHI and result in a significant percent reduction, similar to CPAP, suggesting their role in addressing OSA in non-CPAP adherent patients alongside improving subjective outcomes. Another important point is that AHI as an outcome might not be the best metric to evaluate OSA severity [38]. The comparable reduction in AHI and depression PROMs highlights this fact.

The ESS is a subjective marker for daytime sleepiness and overall sleep quality. While both CPAP and surgical interventions improved ESS scores, both reductions were similar reporting no difference in this subjective outcome between treatments. Our study’s findings are similar to other systematic reviews, which stated that treatment of OSA leads to a reduction in ESS [32, 39, 40], as a marker of daytime sleepiness that subsequently improves patients’ quality of life [41, 42]. 

In this study, our primary goal was to evaluate the impact of sleep surgery on depression that significantly affects patients’ mental health and overall well-being [25]. Intriguingly, both CPAP and surgical interventions demonstrated comparable reductions in depression scores. The comparable improvement in depression scores in both groups prompts a reevaluation of the relationship between physiological improvements and mental health outcomes in OSA management. It suggests that while CPAP might help physiological parameters, surgical interventions can exert a comparable impact on the mental health of individuals with OSA. This could be explained by the longevity of surgery results and CPAP intolerance leading to sleep disruption.

Our study demonstrates that surgical interventions for OSA are not inferior to continuous CPAP therapy, particularly in improving depressive symptoms. Non-inferiority analysis was conducted to compare the effectiveness of both treatments [43]. Notably, the BDI-II scores also improved significantly in both groups, highlighting that the higher mean difference in depression score reduction with surgery did not reach statistical significance (*p* = 0.55). These findings advocate for considering surgical interventions as a viable alternative to CPAP, particularly for patients who experience difficulties with CPAP adherence with or without mood disorders. The comparable effectiveness in both objective and subjective outcomes underscores the potential of surgical interventions to address both physiological and psychological aspects of OSA, supporting a more individualized and patient-centered approach in clinical practice.

These findings have substantial implications for clinical practice, advocating for a more holistic approach to OSA management that considers all aspects of the condition. Physicians shouldn’t just prioritize improvement in physiologic metrics like AHI and recognize that patient-reported outcomes and mental health affect patients’ symptoms. A higher percent reduction in reduction in depression scores in surgical groups prompts a reevaluation of the hierarchy of treatment modalities, emphasizing the need to tailor interventions to individual patient phenotypes, needs, and preferences. When adherence to CPAP is a challenge, it should be recognized that surgical interventions are potential treatments that can address both subjective and objective aspects of the condition [44–47]. The concept of mean disease alleviation which integrates both efficacy and adherence as a true measure of therapeutic effect, becomes more meaningful here. Of note, while both groups reached a significant reduction in their BDI-II scores, they also achieved more than the minimum clinically important difference known to be more than 17.5% reduction. However, the difference between both groups barely reached that difference (M = 10%) [48]. 

Despite the comprehensive nature of our study, several limitations should be acknowledged. The heterogeneity in surgical interventions, varying follow-up periods after both CPAP and surgery, and differences in depression measurement tools pose challenges in synthesizing results. One limitation of this study is the disproportionate contribution of a single large trial to the analysis of depression scores in the CPAP group. Of the 3488 patients included in the meta-analysis for depression outcomes, 3100 were derived from one study. This concentration of data from a single source introduces potential bias and limits the generalizability of the CPAP findings. The large sample size from this study could disproportionately influence the overall effect size, making the results more reflective of that specific population rather than a diverse representation across multiple studies. To mitigate this, we applied appropriate weighting in the meta-analysis, but we acknowledge that this remains a notable limitation. Future studies should aim to include a broader range of trials with more balanced sample sizes to provide a more comprehensive evaluation of depression outcomes in patients with OSA. In addition, non-specific sleep symptoms might falsely elevate patient scores on depression PROMs. Popular scales, such as the BDI, have questions related to sleep symptoms such as insomnia and fatigue that are common among both OSA and psychiatric conditions. However, BDI has demonstrated high internal consistency and reliability in various medical settings, indicating that it can reliably measure depressive symptoms across different clinical populations [49, 50]. A study by Aloia et al. suggests that the BDI-II scale effectively captures depressive symptoms despite the overlap with somatic symptoms of OSA [50]. Another limitation of our study is the lack of consistent data on CPAP adherence across the included studies. While adherence to CPAP is a critical factor influencing treatment efficacy, especially in improving both AHI and depressive symptoms, it was not uniformly reported, limiting our ability to fully assess its impact on the results. Additionally, baseline variables such as body mass index (BMI), which is known to affect both OSA severity and treatment outcomes, were inconsistently reported across studies. This prevents a more detailed sub-analysis of how BMI or other baseline characteristics may influence the efficacy of CPAP or surgical interventions in reducing depressive symptoms. Future studies should aim to collect and report data on adherence and baseline variables more consistently to better understand their role in treatment outcomes. Other limitations include lack of data on co-morbid conditions, medication use, and hypoxic burden (like oxygen-desaturation index), as these factors can also have an impact on depression. In addition, the CPAP group had more severe OSA at baseline as compared to the surgical group, which could potentially have an impact on the degree of improvement that can be produced in subjective symptoms with long-standing severe OSA. Thus, we added the AHI percent reduction to account for this limitation.

It is important to note that culture significantly influence depression, affecting symptom presentation and the acceptance of mental health diagnoses. Our study included data from various cultural contexts, encompassing Eastern and Western populations. For instance, in Western cultures, depression is commonly characterized by psychological symptoms, whereas in many Eastern cultures, it tends to manifest more through somatic symptoms [51]. Including both cultural perspectives in our study is crucial to enhance its generalizability and applicability across diverse populations.

Future research should explore the impact of specific surgical procedures, stratify by severity of OSA, and evaluate long-term mental health outcomes. This can provide insights into which treatment is more appropriate for certain patients. Furthermore, investigating the role of individual patient characteristics and preferences in treatment outcomes can contribute to developing personalized treatment algorithms and provide more precision medicine in OSA.

## Conclusion

Our SR-MA provides insights into comparing the effectiveness of CPAP and surgical interventions for OSA. Both interventions reported significant objective improvement in AHI, and both interventions exhibit comparable effectiveness in reducing subjective depression scores. More research is needed to characterize the patient population that could benefit from these types of interventions.

## Supplementary information

Below is the link to the electronic supplementary material.

ESM 1 (DOCX 24.4 KB)

## Data Availability

The datasets generated and/or analyzed during the current study are not publicly available but are available from the corresponding author on reasonable request.
