# Supplementary material for: Depressive symptoms after surgical and medical management of OSA: a systematic review and meta-analysis
Source: Sleep Breath. 2024 Dec 27;29(1):64. doi: 10.1007/s11325-024-03235-6 (PMC11671573; doi:10.1007/s11325-024-03235-6)
Supplement: Supplementary file 1 — (DOCX 24.4 KB) [file 11325_2024_3235_MOESM1_ESM.docx]

**Supplementary Table and Figures:**


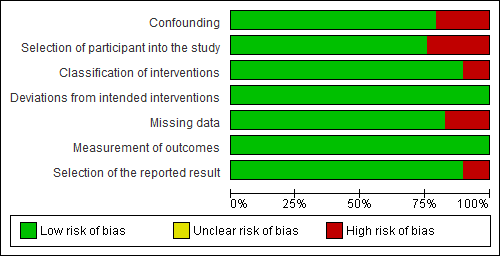


Figure S1 – ROBINS-I Risk of bias graph: review authors' judgements about each risk of bias item presented as percentages across all included studies


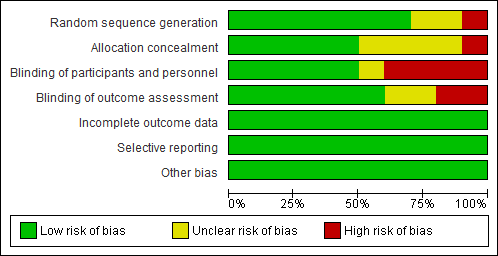


Figure S2 – ROB2 Risk of bias graph: review authors' judgements about each risk of bias item presented as percentages across all included studies

Table S2: PICOTS table

| Element | Description |
| --- | --- |
| Population | Adults with Obstructive Sleep Apnea (OSA) |
| Intervention | Surgical management of OSA |
| Comparison | Continuous Positive Airway Pressure (CPAP) treatment for OSA |
| Outcome | Pre- and post-treatment depression patient-reported outcomes measures (BDI-II) |
| Time | Follow up varies between studies |
| Study Design | Systematic review and meta-analysis |
